# Is it right to look for anti-cancer drugs amongst compounds having antioxidant effect?

**DOI:** 10.1186/2008-2231-20-61

**Published:** 2012-10-20

**Authors:** Mohammad Abdollahi, Seyed Vahid Shetab-Boushehri

**Affiliations:** 1 Department of Toxicology and Pharmacology, Faculty of Pharmacy, and Pharmaceutical Sciences Research Center, Tehran University of Medical Sciences, Tehran, Iran; 2 Department of Medical Nanotechnology, School of Medicine, and Razi Drug Research Center, Tehran University of Medical Sciences, Tehran, Iran

**Keywords:** Cancer, Antioxidants, Free radicals, Oxidative stress

## 

In the recent years, there has been a mounting interest on the use of natural products with antioxidant effect in the management of cancer. If one evaluates search engines will find a burden of papers that have tried to test natural products having anti free radical effects in the management of cancer. But the sense does not seem rational because in cancer we need a compound to kill cancer cells. As a matter of fact, free radicals induce oxidative stress in the body and their deleterious effect in many deliberating diseases that have no exact cure such as diabetes [1], colitis [2], osteoporosis [3], environmental toxins [4] or cancer [5] has been well documented. In spite of this fact, many examples can be given that some although produce free radicals but do not induce cancer such as paraquat toxicity [6] and Alzheimer disease [7]. Superoxide dismutase (SOD) changes superoxide anion radical to hydrogen peroxide which in turn, decomposes to water and oxygen by glutathione peroxidase (GPx) and catalase (CAT). An imbalance between production of free radicals in one side and activities of SOD, GPx and CAT on the other side determines the fate of the cells [4]. Free radicals especially superoxide anion radicals are produced in cancer cells more than what seen in normal cells. Production of free radicals in cancer cells seems a defense mechanism by which body tries to fight against cancer cells. Free radicals especially oxygen species provide a supply for hydrogen peroxide which eventually decomposes to water and oxygen. Oxygen, in turn, reduces neovascularization and metastasis [8]. Thus, cancer is a condition which is accompanied with a decrease in intracellular oxygen. It look likes that every drug or plant extract which can increase intracellular hydrogen peroxide and further decomposition to water and oxygen may be effective in treatment of cancer [5]. On the other hand, hydrogen peroxide can convert to hydroxyl anion and hydroxyl radical via a process called Fenton reaction. Thus, we need a compound to produce free radicals not scavenge free radicals to overwhelm cancer. Although beneficial role of antioxidants in cancer prevention has been well described, it is still in doubt whether or not antioxidants are effective in treatment of cancer. Theoretically, antioxidants scavenge superoxide anion radical and thus deplete the supply of intracellular hydrogen peroxide which may deteriorate cancer conditions. On the other hand, they scavenge hydroxyl radicals which have beneficial effects.

Therefore, we believe that use of antioxidants during chemotherapy of cancer could diminish benefits of chemotherapy or radiation to the patient by scavenging free radicals. This is because radiation and most of anticancer drugs work by generating free radicals to destroy rapidly-dividing cancer cells. So the logical advice to patients who being treated by chemotherapy or radiation is to not taking antioxidant supplements. Meanwhile, the advice to researchers who look for anticancer or cytotoxic drugs amongst antioxidants is to answer the following question? By what mechanism, do you expect antioxidants to kill cancer cells?

## Authors’ contribution

Both authors contributed equally to the paper. Both authors read and approved the final manuscript.
